# Mitochondrial Extracellular Vesicles: A Novel Approach to Mitochondrial Quality Control

**DOI:** 10.3390/biom15081145

**Published:** 2025-08-08

**Authors:** Jie Kong, Rui Sun, Chengying Du, Yiyang Tang, Chengzhi Xie, Qian Li, Li Lin, Hongyan Wang

**Affiliations:** 1 Department of Periodontology, School of Stomatology, China Medical University, Nanjing North Street 117, Shenyang 110000, China; 2022121946@cmu.edu.cn (J.K.); sunrui@cmu.edu.cn (R.S.); 2023122033@cmu.edu.cn (C.D.); 2021353214@cmu.edu.cn (Y.T.); liqian@cmu.edu.cn (Q.L.); 2 Department of Clinical Medicine, Dalian Medical University, Lvshun South Road No 9, Dalian 116044, China; 19982031@cmu.edu.cn

**Keywords:** mitochondria, extracellular vesicle, mitochondrial quality control, MitoEVs, intercellular material exchange, signal transmission

## Abstract

Mitochondria are central to cellular energy metabolism and play a key role in regulating important physiological processes, including apoptosis and oxidative stress. Mitochondrial quality control has recently garnered significant attention, with the underlying mechanisms traditionally considered to be mitophagy and its dynamics. Various studies have demonstrated that extracellular vesicles are crucial for the transmission of mitochondria and their components. These vesicles effectively transport mitochondria to target cells, facilitating intercellular material exchange and signal transmission, thereby enhancing cellular function and viability. This review explores the mechanisms of mitochondrial transfer through mitochondrial extracellular vesicles (MitoEVs), analyzes the novel roles of MitoEVs in mitochondrial quality control, and discusses their applications in disease treatment. We aim to provide new perspectives for future research and support the development of relevant therapeutic strategies.

## 1. Introduction

Mitochondria are considered the cell’s “powerhouse” [1], generating ATP through oxidative phosphorylation (OXPHOS), which provides most of the cell’s energy. Moreover, mitochondria play a vital role in maintaining calcium ion homeostasis [2], regulating the cell cycle, and initiating cell death [3]. Therefore, maintaining mitochondrial homeostasis is essential as imbalances can cause various diseases [4]. Mitochondrial quality control (MQC) is a comprehensive system that regulates the function and dynamics of mitochondria. MQC maintains mitochondrial number, morphology, and function through processes such as mitochondrial fission and fusion, mitophagy, and biogenesis, even under challenging conditions such as hypoxia [5] and inflammation [6].

Recent studies have uncovered the novel function of extracellular vesicles (EVs) in MQC. EVs serve as a “metabolic waste disposal system” for mitochondria by selectively packaging and expelling damaged mitochondria, aberrant mitochondrial DNA (mtDNA), or oxidized proteins. These nanoscale vesicles, ranging from 30 to 1000 nm in diameter, are actively secreted by parental cells into the extracellular space [7]. They are widely distributed in bodily fluids such as blood, saliva, and sweat [8,9,10]. EVs are enclosed by a lipid bilayer and contain bioactive cargoes, including proteins, nucleic acids [11,12], metabolites [13], and other materials originating from the parent cell. Some researchers refer to them as “cargo-delivery packets” and “signalling platforms” [11].

EVs not only remove cellular waste through exocytosis but also facilitate intercellular communication [11]. Released EVs can bind to target cells through specific or non-specific adsorption and deliver their cargo through mechanisms such as endocytosis, micropinocytosis, or plasma membrane fusion [14,15]. This allows EVs to reprogram target cells and regulate their metabolism [16], immune responses [17], and other functions.

Research has indicated that EVs are capable of not only delivering molecules but also transferring entire organelles. EVs that contain intact mitochondria or mitochondrial components are known as mitochondrial EVs. MitoEVs mediate the transmission of mitochondria and their components between cells, regulating the metabolic state of recipient cells. Depending on their source, MitoEVs can have either proinflammatory or anti-inflammatory effects. They may transfer damaged mitochondria, impairing mitochondrial function in recipient cells, or, conversely, repair damaged cells. MitoEVs play diverse roles, offering potential for diagnostic and therapeutic applications, and may serve as novel drug delivery systems. This review provides a comprehensive overview of the origin and function of MitoEVs, with a focus on their role in MQC and their implications for disease.

## 2. Classical MQC

MQC is a complex system that monitors and maintains mitochondrial network function, protecting mitochondria from damage and preventing the accumulation of defective mitochondria.

### 2.1. Mitochondrial Biogenesis

Mitochondrial biogenesis is a highly regulated process that controls the number and size of mitochondria by coordinating the expression of nuclear and mitochondrial genes [18]. This process requires a sophisticated mechanism to ensure accurate transcription and translation of these genes and a system to import nuclear-encoded proteins to maintain mitochondrial morphology and function. Transcription factors, such as peroxisome proliferator-activated receptor-γ coactivator (PGC-1α) and nuclear factor E2-related factor 2 (Nrf2), play critical roles in regulating this process [19,20,21,22], thereby increasing the expression of mitochondrial transcription factor A (TFAM). This enhances intracellular ATP levels and OXPHOS and facilitates the ability of mitochondria to meet metabolic demands and respond to environmental stress [23]. Mitochondrial biogenesis also involves the regulation of gene expression, synthesis of mitochondria-related proteins, and enhancement of cellular energy metabolism and antioxidant capacity [24].

Additionally, upstream regulatory factors can influence this process; for instance, a recent study demonstrated that NAD+, a key substrate for sirtuin deacetylase enzymes, regulates the Sirt1/PGC-1α pathway. Notably, modulating NAD+ levels influences sirtuin activity and, consequently, the Sirt1/PGC-1α pathway [25].

### 2.2. Mitochondrial Fusion and Fission

Mitochondria maintain their functionality and adapt to changing stress conditions through continuous fusion and fission. This process, known as mitochondrial dynamics, eliminates unhealthy mitochondria and prevents their accumulation.

Mitochondrial fusion is the integration of two mitochondria to form a single one. This process can occur at the ends or along the sides of mitochondria, without restriction on their relative position. Given the double-membrane structure of mitochondria, fusion involves outer membrane fusion, mediated by mitofusin 1 and 2, and inner membrane fusion, mediated by Opa1. A time lag may exist between these two events [26]. Mitochondrial fusion regulates mtDNA [27], prevents the accumulation of damaged mitochondrial components, preserves mitochondrial functional integrity, and increases ATP production [28].

Mitochondrial fission is the splitting of one mitochondrion into two. Fission is critical for maintaining the mitochondrial network and regulating cellular energy metabolism. The process is mediated by the cytoplasmic protein DRP1, which translocates to the outer mitochondrial membrane (OMM) upon activation. DRP1 interacts with receptors to promote fission; it assembles into a ring-like structure around mitochondria, constricting them to create new organelles [29].

Mitochondrial fission was previously thought to randomly occur along the mitochondrial axis. However, recent studies have identified other distinct forms of fission: mid-zone and peripheral fission [30]. Additionally, a newly discovered type of fission, known as tail autotomy fission, has been described [31]. These findings offer novel insights into mitochondrial fission and provide new avenues for understanding and treating related diseases.

### 2.3. Mitochondrial Protein Homeostasis

The various functions of mitochondria depend on their internal protein network, which must maintain a dynamic balance during processes such as protein synthesis, folding, modification, and degradation to ensure mitochondrial protein (MP) homeostasis and normal mitochondrial function. Mitochondrial proteases play a crucial role in maintaining this homeostasis, not only by mediating the degradation of misfolded or damaged proteins but also by regulating mitochondrial function through highly regulated proteolytic reactions [32]. Four main ATP-dependent proteases perform protein surveillance: intermembrane AAA protease, matrix AAA protease, Lon protease homologue (LONP), and ATP-dependent Clp protease proteolytic subunit. These proteases generate peptides that are exported to the cytoplasm or further degraded into amino acids by the protease PITRM1. Recent studies have indicated that the MP degradation systems, particularly that mediated by the protease LONP1, plays a role in regulating the fate transition of white adipocytes. This finding suggests that LONP1 could potentially treat metabolic disorders related to adipocyte cell fate programming by restoring MP homeostasis [33].

### 2.4. Mitophagy

External stresses, such as cellular damage, reactive oxygen species (ROS) exposure, or nutrient deprivation, can damage mitochondria, causing them to depolarize and lose their membrane potential. Mitophagy is the process by which damaged mitochondria are selectively enclosed into autophagosomes and fused with lysosomes for degradation to maintain mitochondrial homeostasis [34]. Mitophagy is categorized into two pathways based on the initiating factors and mitophagosome formation mechanisms: the ubiquitin-dependent and the ubiquitin-independent pathways [35].

Of the ubiquitin-dependent pathways, the PINK1/Parkin pathway is one of the most extensively studied [36]. PINK1 is a highly conserved MP involved in regulating mitochondrial function. PINK1 is directed by a mitochondrial-targeting sequence to the inner mitochondrial membrane, where it is cleaved by proteases in the mitochondrial matrix and inner membrane. The cleaved fragments are then secreted into the cytoplasm for degradation by the ubiquitin–proteasome system. However, when mitochondria are damaged or depolarized, PINK1 cannot enter the mitochondria, leading to the accumulation of full-length PINK1 on the OMM. This accumulation recruits and phosphorylates Parkin, activating its E3 ligase activity. The activated Parkin promotes the ubiquitination of mitochondrial surface proteins, marking the damaged mitochondria for degradation by autophagosomes [35,37].

The ubiquitin-independent pathway primarily involves autophagy receptor-mediated mitophagy. These autophagy receptors, located on the OMM, include proteins that can directly interact with LC3 to initiate mitochondrial autophagy [38].

Recent research has revealed additional non-canonical mechanisms for clearing and recycling mitochondria, some of which are classified as type 3 mitophagy [39]. In this process, damaged mitochondrial components are removed in the form of mitochondria-derived vesicles (MDVs). A portion of the damaged mitochondria is enclosed by a membrane, forming vesicles with structures similar to autophagosomes. These MDVs are then released from the mitochondria through budding and are transported to lysosomes for degradation. This mechanism allows the rapid clearance of damaged but non-depolarized mitochondria, contributing to cellular health. Although the exact mechanism behind MDV formation requires further exploration, PINK1 and Parkin participate in the biological initiation of MDVs [40]. The discovery of this MDV degradation pathway provides critical insights into MQC mechanisms and offers new opportunities for therapeutic approaches targeting mitochondrial function and mitochondria-related diseases [41].

## 3. MitoEVs Participate in MQC

### 3.1. Role of MitoEVs Biogenesis and Sorting in MQC

In 1967, Wolf et al. first observed EVs using electron microscopy [42]. Initially, EVs were considered non-functional cellular debris released during reticulocyte maturation. However, subsequent research demonstrated that EVs possess biological functions, such as transporting proteins and mediating intercellular communication [43].

**Figure 1:**
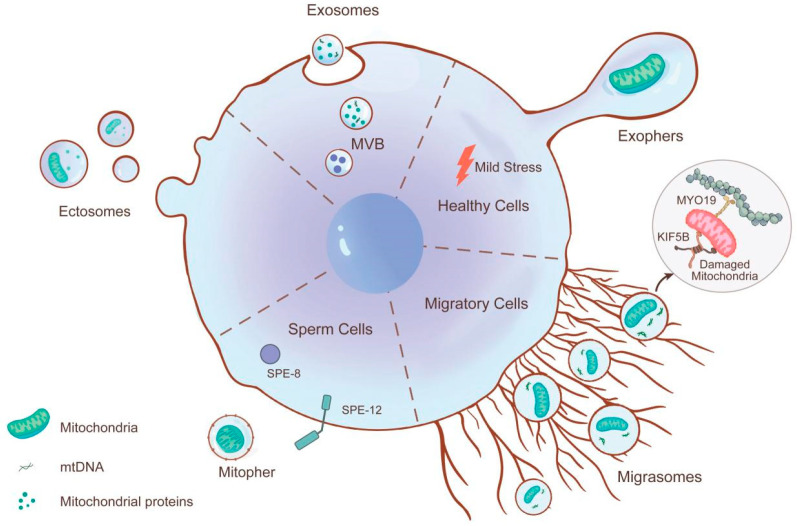
Subtypes of mitochondrial extracellular vesicles (MitoEVs) based on their biogenesis pathways. MitoEVs fall into five subtypes defined by distinct biogenesis: Sperm cells utilize the tyrosine kinase activity of SPE-8 and SPE-12 to transfer individual healthy mitochondria to mitophers. Microvesicles bud directly from the plasma membrane to encapsulate mitochondria. Exosomes originate from the endosomal pathway within multivesicular bodies (MVBs) and are secreted upon MVB fusion with the plasma membrane. Migrasomes mediate mitochondrial expulsion through mitocytosis, a process dependent on motor proteins KIF5B, Drp1, and Myosin19. While under physiological or mild stress conditions, neurons specifically eliminate mitochondria-containing compartments called exophers.

In recent years, the content and functions of EVs have gained considerable attention. In 2024, the International Society for Extracellular Vesicles (ISEV) published the third edition of the international guidelines for EV studies, titled Minimal Information for Studies of Extracellular Vesicles (MISEV2023) [44]. MISEV2023 updates the definition, concepts, origins, and characteristics of EVs, summarizes the related experimental methodologies, and provides guidelines for the release, collection, pre-processing, separation, concentration, and in vivo study of EVs. In accordance with MISEV2023, we classified and described EVs based on their biological origins, physical characteristics (such as diameter and density), and biochemical composition (such as content, markers, presence of mitochondria, and mitochondrial components) (Table 1). The cargo transported by EVs has become a prominent research topic. Mitochondria, which serve as the primary sites of cellular aerobic respiration, have been found in some EVs. Some scholars refer to EVs containing mitochondria and their components as “MitoEVs”. Growing evidence has shown that MitoEVs play a vital role in metabolism, immunity, and inflammation and are closely related to the pathogenesis of various diseases [45]. However, not all EVs contain mitochondria or mitochondrial components. In this review, we have categorized MitoEVs based on the MISEV2023 guidelines and biogenesis pathways (Figure 1).

#### 3.1.1. Exophers

Neurons are the fundamental structural and functional units of the nervous system, communicating with other cells through specialized connections called synapses [56]. Recent studies have detected EVs, namely, exopher around neurons, suggesting their involvement in signal transduction and inter-neuronal communication [48]. The analysis of exophers revealed that under neuronal stress, exophers can transfer harmful substances such as misfolded proteins and damaged mitochondria to neighboring hypodermal cells and more distant coelomocytes for degradation. Additionally, neurons that expel exophers exhibit better functionality, compared with those that do not. Therefore, exophers represent a MQC mechanism that can transport damaged mitochondria out of the cell to maintain neuronal homeostasis, thereby contributing to the preservation of neuronal function. The inhibition of molecular chaperones in neurons leads to increased levels of misfolded proteins and exacerbated mitochondrial damage, thereby increasing the production of exophers.

#### 3.1.2. Migrasomes

Yu et al. first discovered pomegranate-like structures (PLSs) at the trailing ends of migrating cells. These membrane-bound structures, termed migrasomes, contain numerous small vesicles (50–100 nm in diameter) and act as carriers for RNA and proteins, which can be transferred to recipient cells—representing a key source of signaling molecules for intercellular communication [57,58]. Growing evidence indicates that migrasomes are widely present in various cell types [59], tissues [60], and organs [61]. Notably, damaged mitochondria have been identified within migrasomes of L929 cells [62], suggesting that migrasomes serve as MitoEVs and play a role in MQC. Under mild stress conditions, neutrophils transport dysfunctional mitochondria into migrasomes, preventing mitochondrial membrane potential (MMP) collapse and mitochondrial respiration dysfunction, thereby maintaining cellular viability. This process, termed mitocytosis, is mediated by motor proteins such as KIF5B, Drp1, and Myosin19. Importantly, migrasomes not only sequester damaged mitochondria but also selectively remove mutant mtDNA. By clearing dysfunctional mitochondria and mutated mtDNA under stress, mitocytosis safeguards cells from MMP loss and mitochondrial respiratory impairment, reinforcing migrasomes as a critical component of MQC. Therefore, migrasome-mediated mitocytosis represents a novel extracellular MQC mechanism, ensuring mitochondrial homeostasis and cellular health under stress conditions.

#### 3.1.3. Ectosomes

Ectosomes, also referred to as microparticles, microvesicles (MVs), or shedding vesicles, are released from the cell’s surface membrane at a size of 100–1000 nm. These vesicles originate from outward budding of the plasma membrane and separate to form vesicles [51]. Ectosomes contain mitochondria [63] and have been identified as a type of MitoEV. As carriers of mitochondria and components of the MQC system, ectosomes mediate intercellular mitochondrial transfer, thereby regulating cellular energy metabolism and function. They facilitate mitochondrial transfer, particularly in adipose tissue [64]. Research indicates that white adipose cells can transfer mitochondria to macrophages, altering their phenotypes and promoting fat accumulation. Similarly, some studies have identified EVs containing MPs from brown adipose tissue (BAT) and T37I brown adipose cells, indicating that mitochondria adjust their metabolism to enhance thermogenesis in response to cold stimulation. Mitochondria transferred through ectosomes integrate into the mitochondrial network of recipient cells, leading to raised levels of AMP, ATP, and oxidative species, and this promotes the metabolic function of the recipient cells. Similarly, during oxidative stress, macrophage uptake depolarizes mitochondria released by human mesenchymal stem cells (MSCs) through ectosomes, which enhances ATP production and reduces mitochondrial ROS. This process maintains macrophage homeostasis and function, thereby serving as a pro-survival mechanism [65].

#### 3.1.4. Exosomes

Exosomes, with diameters of approximately 30–150 nm, are nanoscale spherical lipid bilayer vesicles secreted by cells [51]. Due to their small size, exosomes cannot enclose intact mitochondria; however, multiple studies have demonstrated that exosomes contain molecules associated with mitochondria, such as cardiolipins, mtDNA, and MPs [66]. Considerable evidence suggests that mtDNA transported by exosomes can participate in disease processes [67]. In vitro experiments have shown that pyroptotic cells release mtDNA through exosomes, a process activated by Caspase-1 and Gasdermin-D. In vivo studies indicate that the mtDNA encapsulated within these exosomes can be released; this triggers the production of pro-inflammatory factors such as IL-1β and IL-23 through mechanisms mediated by NLRP3 and TLR9, thereby promoting an inflammatory response in Behçet’s syndrome (BS) [68]. Xia et al. revealed that adipose-derived AdMSC-Exos can effectively transfer mtDNA and other mitochondrial components to alveolar macrophages in a dose-dependent manner [69]. This transfer improves macrophage mitochondrial integrity and phosphorylation levels, promotes a shift towards an anti-inflammatory phenotype, restores macrophage metabolism, and alleviates lung inflammation.

#### 3.1.5. Mitopher

Tang et al., using TEM, observed a novel vesicular structure containing mitochondria in the sperm cells of male *Caenorhabditis elegans* [53]. This structure rapidly forms after sperm cell formation through outward budding of the plasma membrane. Researchers named this specific EV that encapsulates mitochondria a “mitopher” and referred to the process of its formation as “mitopherogenesis”. Each mitopher contains only one healthy mitochondrion, which is a distinguishing feature of mitophers and plays a crucial role in intercellular mitochondrial, genetic material exchange, and cell signal communication. When the generation of a mitopher is suppressed, mitochondria accumulate in sperm cells, and sperm motility is also impaired. Therefore, it is proposed that the production of mitophers is a key mechanism regulating mitochondrial quantity in the sperm. The formation of mitophers is considered a novel pathway for expelling healthy mitochondria, which may act as part of the MQC mechanism to eliminate excess mitochondria. The tyrosine kinases SPE-8 and SPE-12 partially mediate extracellular protease-induced mitopherogenesis.

### 3.2. MitoEVs Involved in the Transfer of Mitochondria

**Figure 2:**
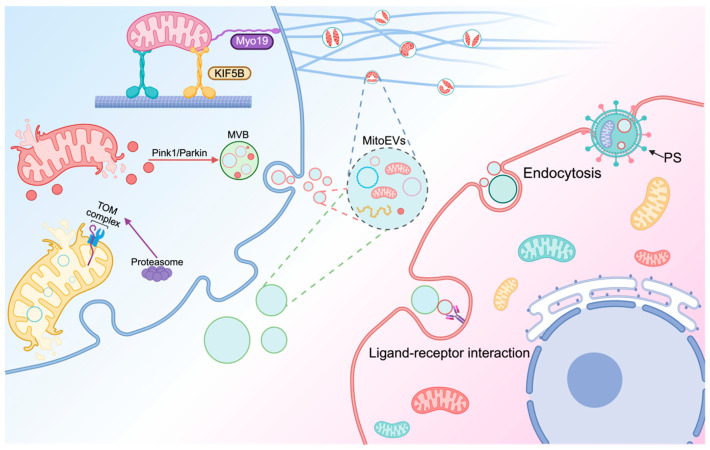
Schematic overview of mitochondrial cargo sorting into EVs and their specific recognition by recipient cells. The PINK1/Parkin pathway ubiquitinates damaged mitochondria for MitoEV packaging, while motor proteins (KIF5B and dynein) and Drp1 facilitate mitochondrial transport. The TOM complex mediates selective cargo loading (e.g., cytochrome C and mtDNA-binding proteins). MitoEVs bind recipient cells via surface ligands and phosphatidylserine (PS)-mediated recognition. Endocytes occur primarily through clathrin-mediated endocytosis, with minor roles for macropinocytosis and caveolae-dependent uptake.

EV-mediated mitochondria perform two key functions [70]. Healthy mitochondrial components and whole mitochondria can be transmitted to recipient cells through EVs, serving as a “rescue” function [71]. In contrast, damaged mitochondrial components and mitochondria can be expelled extracellularly through EVs and cleared by other cells through pathways such as the mitochondrial–lysosomal axis, serving as a “clean-up” function [72]. The selective incorporation of mitochondrial cargos into extracellular vesicles (EVs) involves multiple mechanisms (Figure 2), and one of the key pathways is the activation of the PINK1/Parkin signaling. Under stress, PINK1 accumulates on depolarized mitochondria, phosphorylates Parkin, and recruits E3 ubiquitin ligases to ubiquitinate membrane proteins like VDAC1, thereby labeling damaged components for MitoEV packaging [73,74]. Motor proteins such as KIF5B and dynein facilitate cargo sorting through mechanical transport; they move damaged mitochondria to the plasma membrane, link them to actin filaments, and enable Drp1-mediated fission into migratory bodies, which subsequently form MitoEVs [62]. Additionally, the TOM complex mediates precise cargo selection by recognizing specific sequences on target proteins. It guides the incorporation of proteins such as cytochrome C and mtDNA-binding proteins into MitoEVs, a process critical for intercellular mitochondrial signaling and function regulation. These mechanisms operate to ensure the selective packaging of mitochondrial cargo into MitoEVs [75].

The precise mechanisms through which MitoEVs bind to recipient cells remain unclear. However, as a type of EVs, their interaction process is expected to share similarities with general EV mechanisms, primarily involving recognition and uptake [76]. The surface of MitoEVs is rich in various membrane proteins, lipids, and glycans, which act as ligands. These ligands enable specific recognition and binding with receptors on the recipient cell surface, such as integrins or heparan sulfate proteoglycans, initiating the capture of MitoEVs by target cells [77]. Phosphatidylserine (PS) is expressed on the surface of various MitoEVs, which is a common “eat me” signal. For example, during cardiac stress, cardiomyocytes eject damaged mitochondria through exophers. The Mertk receptor on macrophages binds to PS, thereby enabling the fusion with macrophages to promote the restoration of mitochondrial function in cardiomyocytes [78]. Meanwhile, endocytes also play a role in MitoEV fusion. Activated platelets enhance the wound-healing capacity of MSCs by transferring respiratory-competent mitochondria through MitoEVs. This mitochondrial transfer is predominantly mediated by dynamin-dependent clathrin-mediated endocytosis, with macropinocytosis and caveolae-mediated endocytosis playing minor roles in the internalization process [79].

Herein, we introduce two types of EV-mediated mitochondrial transfer systems and their significance.

#### 3.2.1. MitoEVs Act as Rescuers by Transferring Healthy Mitochondria to Damaged Cells

EV-mediated mitochondrial transfer has emerged as a novel cell-free therapy [17]. Donor cells can transport healthy mitochondria to damaged cells through EVs, restoring cellular function and providing therapeutic effects [80]. Among these, MSCs exhibit robust self-renewal, differentiation, repair capabilities, and transplantability [81]. They have significant potential in therapeutic tissue engineering and regenerative medicine. Currently, “stem cell therapy” is being applied to treat various diseases. Stem cells can receive “distress signals” and transfer their own material, including mitochondria, to other cells to provide assistance. Therefore, stem cells often act as donor cells for mitochondrial transfer [70].

Neutrophil extracellular traps (NETs) are web-like structures produced by neutrophils in response to stimulation and extend into the extracellular space. These NETs capture bacteria, facilitate immune responses [82], and are associated with inflammation and disease progression [17]. EVs derived from human umbilical cord MSCs (hUC-MSCs) transmit healthy mitochondria to neutrophils, enhance mitochondrial function, and reduce NET production. Additionally, the injection of hUC-MSC-EVs into ischemia–reperfusion injury (IRI) mice through the tail vein significantly reduced NET formation in the liver, demonstrating a protective effect against liver injury. In summary, hUC-MSC-EVs facilitate mitochondrial transfer to neutrophils, improving their function and providing therapeutic benefits for liver IRI.

Similarly, MSCs can also receive “distress signals” and transfer healthy mitochondria to macrophages, enhancing their anti-inflammatory and phagocytic functions and thus reducing lung injury [83]. This transfer of healthy mitochondria acts as a “rescue” mechanism, regulating mitochondrial quality within macrophages.

#### 3.2.2. MitoEVs as Cleaners by Excluding Damaged Mitochondria

Cells interact in various ways within the body, and this collaboration ensures the normal functioning of the internal environment. Macrophages, with their strong phagocytic abilities, often serve as endpoints for the transport of intercellular materials [84]. As mediators of intercellular communication, EVs can transfer damaged mitochondrial components to macrophages. Macrophages identify these components by interacting with specific pattern recognition receptors, which recognize damage-associated molecular patterns. The macrophages then degrade and digest foreign materials through lysosomes, ultimately clearing waste and acting as “garbage collectors”. In this way, they are involved in the maintenance of mitochondrial quality and stability [85].

For example, brown adipose tissue (BAT) cells encapsulate oxidatively damaged mitochondria in EVs under cold conditions. BAT macrophages clear these mitochondria, purifying the metabolic environment within BAT cells. This process enhances the thermogenic function by controlling mitochondrial quality [77]. Additionally, Phinney et al. reported that MV-mediated mitochondrial transfer under oxidative stress improves the survival of recipient macrophages by enhancing their mitochondrial bioenergetics [65].

Furthermore, researchers have discovered exophers around the body wall muscles (BWMs) of *C. elegans*. The process of generating these vesicles is referred to as “exopheresis” [86]. Using electron microscopy to observe their contents, researchers found that exophers in BWMs contain diverse mitochondria. Therefore, they speculate that exophers transport damaged mitochondria to the extracellular space, representing a novel EV-mediated mechanism for MQC. This process impacts the signaling and exchange of mitochondria and mitochondrial genetic material between cells. Researchers suggest that exopheresis is a novel mechanism of MQC that could influence reproductive system function through mitochondrial transfer.

**Figure 3:**
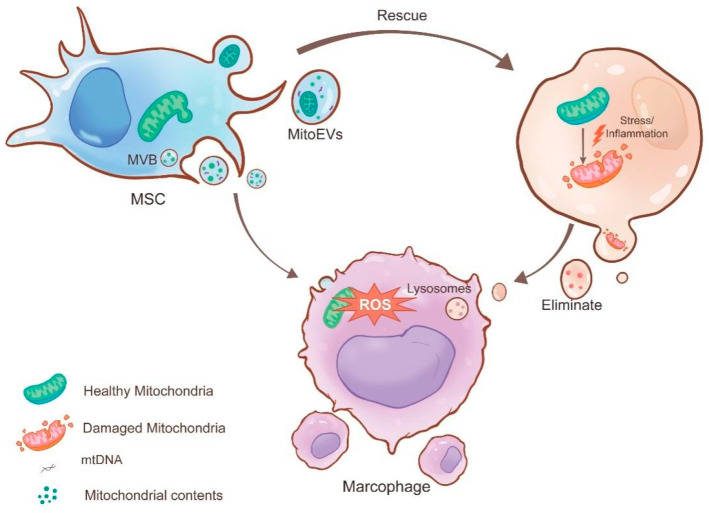
Mesenchymal stem cells (MSCs) serve as donor cells, transferring healthy mitochondria and their components to damaged recipient cells via MitoEVs to maintain cellular homeostasis. Similarly, donor cells impaired by external stimuli also transfer their damaged mitochondria and components to macrophages through MitoEVs. The macrophages then degrade and process these impaired mitochondria via lysosomes, thereby maintaining mitochondrial quality control (MQC) within the cells.

Similarly, researchers have found that exopheresis, a process involved in MQC in the heart, allows cardiomyocytes to transfer damaged mitochondria to cardiac-resident macrophages (cMacs) [78]. This process is driven by components of the autophagy mechanism and relies on exosomes to transfer damaged mitochondria to healthy cMacs in the heart. cMacs phagocytize these mitochondria from cardiomyocytes, and when cMacs are depleted, the expression of mitochondria-related proteins in cardiomyocytes significantly decreases, while the number of mitochondria increases. Additionally, mitochondrial morphology changes, with a reduction in crista density and an increase in mitochondrial area. cMacs influence cardiac function by regulating mitochondrial quantity and quality in cardiomyocytes Figure 3.

### 3.3. MitoEVs Transfer Mitochondrial Components

EVs not only transfer intact mitochondria but also maintain mitochondrial network homeostasis by transporting mitochondrial-related components. Some studies have demonstrated that exosomes can transfer mtDNA. For example, alveolar macrophages can take up exosomes from adipose-derived stem cells, resulting in the transfer of mtDNA and the increased expression of M2 macrophage markers, which alters their phenotype and function. When the mtDNA in these exosomes is depleted, the therapeutic effect diminishes [69]. Similarly, Liu found that stem cells transport mtDNA from exosomes to recipient cells through TFAM, thereby restoring mtDNA and TFAM levels in target cells and maintaining mitochondrial homeostasis under oxidative stress [87]. The transfer of mitochondrial components primarily relies on exosomes as intercellular communication vehicles. The supplementation of damaged mitochondria enhances mitochondrial ATP production and OXPHOS activity, providing an effective means of controlling mitochondrial quality.

### 3.4. Cross-Talk Between Different Mechanisms of MQC

Classical MQC involves several cellular mechanisms aimed at maintaining mitochondrial function and integrity, including mitochondrial fusion and fission, mitophagy, and mtDNA repair. These mechanisms eliminate damaged mitochondria and preserve mitochondrial health. Mitochondrial transfer delivers fully functional mitochondria to compensate for damaged ones and support mitochondrial function, and this process is associated with classical MQC. It has been reported that mitochondrial biogenesis promotes mitochondrial transfer [88]. In a mouse model of pulmonary fibrosis, mitochondrial biogenesis enhances the transfer of mitochondria from human MSCs to lung epithelial cells injured by bleomycin. Similarly, artificial mitochondrial transplantation can induce autophagy mediated by the PINK1/Parkin pathway, further enhancing mitochondrial biosynthesis. This suggests a mutually reinforcing relationship between mitochondrial transfer and autophagy [89]. Additionally, mitochondrial transfer is closely linked to MDVs [77]. Under oxidative stress, with ROS levels rising, BAT cells form PINK1-mediated oxidatively damaged mitochondria, which generate PDHβ + TOMM22-MDV to expel oxidized proteins. MDVs transfer these proteins to EVs, which release them extracellularly to ensure MQC [62]. This indicates that mitocytosis and mitophagy are coordinated. The interaction between mitochondrial transfer and classical MQC mechanisms maintains cellular homeostasis, ensuring the removal of damaged mitochondria while redistributing healthy ones to support cellular functions. Understanding these processes will provide insights into new therapeutic strategies for improved mitochondrial health and function towards the treatment of various diseases.

### 3.5. Mitochondrial Transfer Through Other Pathways

In addition to the aforementioned mechanisms, mitochondrial transfer can also occur through other pathways, including free mitochondrial transfer, tunneling nanotube (TNT)-mediated transfer, and gap-junction-related mechanisms. Free mitochondrial transfer refers to the release of mitochondria that are not enclosed by any membrane structures (such as EVs). These free mitochondria can be taken up by neighboring cells through phagocytosis or other mechanisms. They exhibit diverse origins, intact structures, and fully functional activity. Recent studies [80] have detected structurally intact and functionally active free mitochondria with complete respiratory chain activity in human blood under normal physiological conditions. However, their precise roles in both physiological and pathological states remain under investigation. TNT-mediated mitochondrial transfer involves long, thin intercellular bridges that facilitate the transfer of mitochondria and other organelles between cells. Unlike EVs, this mode of transport is bidirectional. Saha et al. [90] demonstrated that cancer cells can “hijack” mitochondria from immune cells through physical nanotubes. This TNT-mediated transfer not only enhances the metabolic capacity of cancer cells but also leads to mitochondrial depletion in immune cells. Gap-junction-dependent adhesion represents another contact-dependent transfer mechanism involving connexin 43 (Cx43), which we define as “adhesion-mediated mitochondrial transfer”. Although the pores of gap junction channels are too small to allow direct mitochondrial passage [91], gap junctions may indirectly influence mitochondrial function. Cx43 could promote TNT formation by recruiting actin remodeling, thereby facilitating intercellular mitochondrial transfer [92,93].

## 4. Clinical Translation of MitoEVs

**Figure 4:**
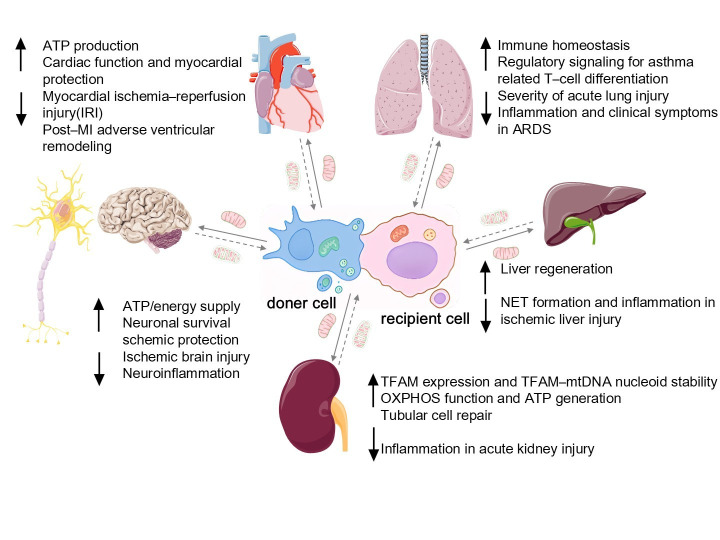
Mitochondrial transfer and disease regulation mechanisms. Donor cells donate healthy mitochondria to damaged cells in different organs. This process can restore ATP production, correct oxidative stress, and induce metabolic remodeling or immunomodulatory effects, thereby playing a therapeutic role in various diseases (such as inflammation, heart disease, and lung diseases). Meanwhile, damaged cells actively eliminate dysfunctional mitochondria through the MQC system, preventing their accumulation, which could otherwise lead to apoptosis or exacerbated inflammation.

The biological functions of MitoEVs and their role in intercellular signaling are closely tied to clinical systemic diseases (Figure 4). On one hand, MitoEVs from damaged cells can have a pathological impact on various diseases by inducing ROS bursts, mitochondrial defects, and other harmful mechanisms in recipient cells. On the other hand, MitoEVs also show promise as therapeutic agents in various systems owing to their metabolic or immunomodulatory effects (Table 2).

### 4.1. Cardiovascular System

Cardiovascular diseases represent a significant global health challenge, with mitochondria playing vital roles in heart function and energy metabolism. Therefore, MitoEVs present a novel therapeutic approach for treating cardiovascular conditions. Crewe et al. discovered a new mechanism, interorgan mitohormesis [95], which involves the transfer of damaged mitochondria to recipient cells by sEVs. This process induces adaptive responses in the recipient cells and establishes a self-protective mechanism. In mouse models, signals from fat cells protect the heart from the damage caused by obesity and limit myocardial IRI. Additionally, researchers have observed that mitochondrial transfer through MVs into induced cardiomyocytes (iCMs) activates mitochondrial biogenesis in recipient iCMs, significantly improving cardiac function in mice following myocardial infarction (MI) [96]. However, after myocardial infarction, cardiac fibroblasts can transfer damaged mitochondrial components through MitoEVs, promoting macrophage inflammatory activation and exacerbating maladaptive ventricular remodeling through NLRP3 activation. Inhibition of CFs-mt-sEV and NLRP3 can improve cardiac function and attenuate post-MI ventricular remodeling [107]. MVs also promote the transfer of both mitochondrial and non-mitochondrial cargo, aiding intracellular energy improvement in vitro.

Furthermore, MVs transfer healthy mitochondria into endothelial cells [94], increasing ATP levels by 100–200 folds. This enhances endothelial cell survival and offers a mechanism for brain ischemia protection, which could be used in treating ischemic stroke. The protective effects of MitoEVs against MI and brain ischemia highlight their potential to treat ischemic diseases and paves the way for new cardiovascular treatments.

### 4.2. Nervous System

Mitochondria can exert neuroprotective effects through various mechanisms, including maintaining calcium homeostasis and regulating ROS levels. In a brain ischemia model, astrocytes release MitoEVs to protect neurons from hypoxia and glucose deprivation [98]. Researchers have found that macrophages transmit mitochondria to sensory neurons through the release of EVs to actively alleviate inflammatory pain [97]. Pain relief and mitochondrial transfer are mediated by the interaction between M2 macrophages and the CD200R/iSec1 receptor–ligand complex in neurons, which facilitates the docking of EVs with sensory neurons, enabling mitochondrial transfer and alleviating inflammatory pain. In some degenerative neurological diseases, such as multiple sclerosis [99], neural stem cells (NSCs) release EVs containing mitochondria that are transferred to monocytes, restoring their mitochondrial dynamics and cellular metabolism while reducing the expression of pro-inflammatory markers in target cells. In animal models, exogenous NSCs transfer mitochondria to monocytes and significantly improve clinical defects. These novel therapeutic strategies focus on restoring neuronal mitochondrial homeostasis or enhancing mitochondrial transfer to macrophages.

### 4.3. Respiratory System

In the respiratory system, MitoEVs reduce inflammation in pulmonary diseases by transferring mitochondria or their components. Researchers have found that pro-inflammatory macrophage-derived EV exosomes transfer mitochondria to recipient cells, altering the pro-inflammatory functions and signaling capabilities of these organelles [101]. This improved retrograde signal transduction can regulate the metabolic changes of target T cells and ultimately change the differentiation and function of T cells in chronic inflammatory diseases, such as asthma. Additionally, MSC-derived EVs can improve the clinical symptoms of acute respiratory distress syndrome by transferring functional mitochondria to regulate the barrier properties and inflammatory responses of primary human pulmonary microvascular endothelial cells and small airway epithelial cells [100]. Mitochondrial components, such as mtDNA, can also be transferred through exosomes to promote macrophage metabolism and immune homeostasis, thereby alleviating the severity of acute lung injury [69].

### 4.4. Immune System

The immune system is the first line of defense in the body and is closely related to overall health. Macrophages engulf damaged mitochondria to maintain cellular homeostasis. Mitochondria can coordinate signaling and effector functions to activate immune cells and antimicrobial defense. Small EVs derived from IFN-γ and TGF-β1-licensed MSCs suppress proinflammatory THP-1 macrophage activation while promoting an anti-inflammatory phenotype, characterized by reduced secretion of TNF-α and IL-1β, along with increased IL-10 production. These EVs also induce a higher proportion of regulatory T cells and exhibit enhanced suppression of allogeneic T-cell proliferation [108]. In oral squamous cell carcinoma, EVs transfer mtDNA, which is released owing to Lon induction. This mtDNA triggers IFN-γ/PD-L1 expression in mouse cells, causing EVs to directly secrete and suppress T-cell activation, thus weakening anti-tumor immunity. These findings suggest that the mtDNA and PD-L1 carried by EVs could be potential diagnostic biomarkers for anti-PD-L1 immunotherapy [103]. Additionally, because exosomes can transmit danger signals between cells without specific receptors, exosomes containing mtDNA from Behçet’s syndrome (BS) cells not only promote inflammatory characteristics in the manifestation of BS, leading to disease onset, but can also induce a strong inflammatory response in adjacent cells. This suggests that exosome-mediated mtDNA secretion is a potential alarm transduction pathway. Therefore, blocking the secretion of mtDNA through exosomes may be a possible way to treat inflammatory diseases [68]. Furthermore, Levoux and colleagues discovered that platelets release fully functional mitochondria, which MSCs uptake through endocytosis [79]. This uptake leads to metabolic reprogramming, increasing citrate levels and enhancing MSC pro-angiogenic function, indicating that MitoEVs can promote tissue repair. Mitochondria and their components, transferred by MitoEVs, are potential therapeutic targets for immune system diseases.

### 4.5. Digestive System

In digestive system diseases, such as ischemic liver injury, the formation of NETs in the inflammatory response may lead to liver damage during the hepatic ischemic phase. Lu et al. discovered that hUC-MSCs release EVs containing healthy mitochondria, which are transmitted to neutrophils in the liver, triggering mitochondria fusion [17]. This process restores the status and function of the mitochondria in neutrophils, thereby reducing NET formation. In addition to transferring healthy mitochondria to suppress inflammatory responses, recent studies have found that EVs in the serum of patients with colon cancer are enriched with structurally intact circular mtDNA [104]. Exogenous mtDNA can be transported into the mitochondria of adjacent colon epithelial cells, causing an improvement in ROS levels. The increasing ROS leads to the translocation of the transcription factor RelA to the nucleus and activates the transcription of TGFβ1, exacerbating the malignant phenotype of colon cancer cells. Similarly, in the liver cells of aldehyde dehydrogenase 2 gene-deficient mice, EVs can transfer harmful oxidative mtDNA to neighboring liver cells, activating multiple carcinogenic pathways and ultimately leading to the development of alcohol-related hepatocellular carcinoma [109]. Pancreatic β-cells internalize inflammatory MitoEVs, whose transferred mitochondria fuse with endogenous networks, inducing lipid peroxidation, organelle collapse, and STING-mediated apoptosis through cytosolic mtDNA release [110].

### 4.6. Urinary System

The current research on EVs in the urinary system is primarily focused on their therapeutic applications. Specific cell-derived EVs, such as those derived from stem cells, are directly used as therapeutic agents because of their bioactive molecules. For example, exosomes derived from MSCs transfer MPs and mtDNA to human renal proximal tubular cell lines to alleviate mtDNA damage and inflammation following acute kidney injury. MitoEVs restore TFAM protein and TFAM-mtDNA complex (nucleoid) stability to enhance TFAM expression, reversing mtDNA loss and mitochondrial OXPHOS defects in damaged renal tubular cells and effectively reducing mitochondrial damage and inflammation in kidney injury cells [87]. Additionally, EVs can serve as delivery vehicles for many drugs used in the treatment of kidney diseases. As carriers of nucleic acid drugs, researchers have found that EVs derived from B cells can transfer harmful miR-3960 to renal tubular epithelial cells over long distances, leading to mitochondrial damage in tubular cells. Targeting EV release or miR-3960 expression may reduce obesity-related kidney damage [106].

### 4.7. Other Systems

MitoEVs also play crucial roles in other systems. In the musculoskeletal system, large MVs derived from MSCs can transmit intact functional mitochondria. To relieve pain associated with osteoarthritis and protect articular cartilage, healthy mitochondria are transmitted to chondrocytes without direct cell-to-cell interactions. This transfer of mitochondria can potentially help to alleviate OA symptoms and maintain joint health [105]. Similarly, MitoEVs play a role in brown adipose tissue [77], and, in response to cold stimulation, BAT releases damaged mitochondria in vesicular form. These oxidatively damaged mitochondria are then cleared by macrophages within the adipose tissue to ensure the thermogenic function of BAT. This process helps maintain the metabolic activity and heat production capacity of BAT. In summary, MitoEVs have diverse functions and applications, highlighting their versatility and therapeutic potential in various physiological systems and underscoring their importance in advancing our understanding of disease mechanisms and potential treatment strategies.

## 5. Conclusions and Future Perspectives

MitoEVs play a novel role in MQC by transferring healthy mitochondria and promoting the recovery of damaged recipient cells. Simultaneously, macrophages engulf damaged mitochondria and their components from these vesicles and fuse them with existing mitochondria to maintain intracellular stability. MitoEV-mediated MQC occurs under both physiological and pathological conditions, making it a new target for the diagnosis and treatment of various diseases. The biological information that they carry can serve as biomarkers for multiple diseases and may also act as carriers for targeted drug therapies. Nevertheless, the naming and characterization methods of EVs lack standardization and require more scientific experimental techniques for isolation and storage. Advances in biotechnology are expected to refine MitoEV properties, enabling the synthesis of next-generation, artificially engineered vesicles with enhanced bioactivity, stability, and targeting capacity—thereby accelerating clinical translation [111]. This article summarizes the types of MitoEVs involved in MQC and the significance of mitochondrial and/or its component transfer between cells. It also discusses the applications of MitoEVs in disease diagnosis and treatment, highlighting their clinical prospects and encouraging further research in this field.

## Figures and Tables

**Table 1:** Types of extracellular vesicles (EVs).

Subtype	Size (nm)	Markers	Biogenesis/Release	Cargo	Refs.
Large oncosomes	1000–10,000	Caveolin 1, CK18, and GAPDH	Cancer cells	Proteins and nucleic acids	[46,47]
Exophers	1000–7800	Phosphatidyl-serine	Jettisoned from cell body	Mitochondria, lysosomes, and protein aggregates	[48]
Apoptotic bodies	1000–5000	CD9, CD63, CD81, C3b, and TSP	Budding from plasma membrane during apoptosis	Mitochondria, ribosomes, and proteins	[49]
Migrasomes	500–3000	TSPAN4, CPQ, EOGT, NDST1, and PIGK	Cell migration	Mitochondria, mtDNA, and proteins	[50]
Ectosomes (microparticles, microvesicles, and shedding vesicles)	100–1000	Annexin A1 and A2, and ARF6	Outward budding of the plasma membrane	Mitochondria, mitochondrial proteins, proteins, lipids, and carbohydrates	[14]
Exosomes	30–150	CD63, CD9, CD81, TSG101, Alix, and HSP70	Originating in the endosomal pathway in the MVB and released when MVB fused with plasma membrane	mtDNA, mitochondria-pertinent components, proteins, glycoconjugates, lipids, nucleic acids, and metabolites	[51,52]
Mitopher	490–1100	Unknown	Outward budding off	One single mitochondrion	[53]
Pyroptotic extracellular vesicles	60–200	ASC and Annexin V	Pyroptotic cells	Unknown	[54]
Blebbisomes	up to 20,000	VDAC2, VDAC1, and TGN protein 2	A single retraction event where a cell fragment remains attached to the substrate via a membrane nanotube and is released upon severing of the nanotube	Mitochondria and cellular organelles	[55]

**Table 2:** Therapeutic potential of mitochondrial extracellular vesicles (MitoEVs) in different systems.

Organ/System/Disease	Donor	Types of MitoEVs	Recipient	Cargos	Mechanism/Effect	Refs.
Cardiovascular System/Brain/Ischemic Stroke	Brain endothelial cell	Microvesicles	Endothelial cells and neurons	Polarized mitochondria	ATP production ↑, endothelial cell survival ↑	[94]
Cardiovascular System/Cardiac/Ischemia–Reperfusion Injury	Adipocyte	Exosomes	Cardiomyocyte	Oxidatively-damaged mitochondrial particles	Induce adaptation in recipient cells, protect the heart from damage caused by obesity	[95]
Cardiovascular System/Cardiac/Ischemic Myocardium	Autologous-stem-cell-derived cardiomyocytes	Microvesicles	Cardiomyocyte	Mitochondria	Mitochondrial biogenesis ↑, cardiac function ↑	[96]
Nervous System/Neuronal/Pain	Macrophages	Microvesicles	Sensory neurons	Mitochondria	CD200R/iSec1 receptor–ligand complex, inflammatory pain ↓	[97]
Nervous System/Neuronal/Cerebral Ischemia	Astrocytes	Microvesicles	Neurons	Mitochondria	Protect neurons from hypoxia and glucose deprivation	[98]
Nervous System/Ventricular/ Degenerative Neurological Diseases	Neural stem cell	Microvesicles	Monocytes	Mitochondria	Restore mitochondrial dynamics and cellular metabolism	[99]
Respiratory System/Lung/Acute Respiratory Distress Syndrome	Mesenchymal stem cells	Microvesicles	Human pulmonary microvascular endothelial cells and human small airway epithelial cells	Functional mitochondria	Barrier integrity of human primary lung epithelial and endothelial cells ↑, symptoms of ARDS ↓	[100]
Respiratory System/Lung/Acute Respiratory Distress Syndrome	Mesenchymal stem cells	Microvesicles	Monocyte-derived macrophages	Functional mitochondria	CD206 expression ↑, associate with ARDS	[83]
Respiratory System/Lung/Acute Lung Injury	Mesenchymal stem cells	Exosomes	Alveolar macrophages	mtDNA	Macrophage metabolism and immune homeostasis ↑, associate with acute lung injury	[69]
Respiratory System/Bronchial/Asthmatic	Airway myeloid-derived regulatory cells	Exosomes	Peripheral T cells	mtDNA	Alter the function of T cells, associate with asthma	[101]
Immune System/Multi-organ/Leukemia	Necroptotic cells	Microvesicles	Macrophage	Healthy mitochondria	Immune activation, inflammation ↓	[102]
Immune System /Oral/Tumor	Oral squamous cell carcinoma cells	Exosomes	Macrophage	mtDNA	T-cell activation ↓, anti-tumor immunity ↓	[103]
Immune System/Multi-organ/Behçet’s disease	Pyroptotic cells	Exosomes	Adjacent cells	mtDNA	Inflammatory response ↑, associate with BS	[68]
Digestive System/Hepatic/Ischemia–Reperfusion Injury	Mesenchymal stem cells	Microvesicles	Neutrophil	Healthy mitochondria	NET formation ↓, associate with liver IRI	[17]
Digestive System/Colon/Colon Cancer	Colon cancer cell	Exosomes	Adjacent colonic epithelial cells	mtDNA	ROS ↑, associate with colon cancer	[104]
Locomotor System/Bone/Regenerative Orthobiologic	Mesenchymal stem cells	Microvesicles	Chondrocytes	Healthy mitochondria	OA symptoms and pain ↓, preserve articular cartilage	[105]
Endocrine System/Adipose Tissue/Thermogenesis	Brown adipocytes	Microvesicles	Macrophages	Damaged mitochondria	Releases damaged mitochondria, restore thermogenic function	[77]
Urinary System/Kidney/Acute Kidney Injury	Mesenchymal stem cells	Exosomes	Renal proximal tubular cell lines	Mitochondrial proteins, mtDNA	TFAM expression ↑, treat kidney injury	[87]
Urinary System/Kidney/ Obesity-related Kidney Injury	B Lymphocyte	Exosomes	Proximal tubule epithelial cells	miR-3960	Mitochondrial damage ↑, associate with obesity-related kidney damage	[106]
Reproductive System	Sperm cells	Mitopher	Unknown	Mitochondrion	Regulates sperm mitochondrial quantity and fertility	[53]
Multiple Systems	Migratingcell	Migrasome	Unknown	Damaged mitochondria	Remove damaged mitochondria and maintain cell viability	[62]
Cardiovascular System	Cardiomyocytes	Exophers	Macrophages	Dysfunctional mitochondria	Maintain cardiomyocyte health and cardiac function	[78]

↑: produce, ↓: reduce. In summary, MSCs and adipocyte serve as the donor cells for mitochondrial MitoEVs, while macrophages, cardiomyocytes, and neurons act as recipients via MitoEV uptake.

## Data Availability

Data sharing is not applicable to this article as no datasets were generated or analyzed during the current study.

## Abbreviations

MitoEVs	mitochondrial extracellular vesicles
OXPHOS	oxidative phosphorylation
MQC	mitochondrial quality control
EVs	extracellular vesicles
PGC-1α	peroxisome proliferator-activated receptor-γ coactivator
Nrf2	nuclear factor E2-related factor 2
TFAM	mitochondrial transcription factor A
mtDNA	mitochondrial DNA
OMM	outer mitochondrial membrane
MP	mitochondrial protein
ROS	reactive oxygen species
TNT	tunneling nanotube
Cx43	connexin 43
MDVs	mitochondria-derived vesicles
MSCs	mesenchymal stem cells
MVs	microvesicles
BS	Behçet’s syndrome
MVB	multivesicular body
NETs	neutrophil extracellular traps
hUC-MSCs	human umbilical cord MSCs
IRI	ischemia–reperfusion injury
BAT	brown adipose tissue
cMacs	cardiac-resident macrophages
iCMs	cardiomyocytes
NSCs	neural stem cells
